# A brain-computer interface with vibrotactile biofeedback for haptic information

**DOI:** 10.1186/1743-0003-4-40

**Published:** 2007-10-17

**Authors:** Aniruddha Chatterjee, Vikram Aggarwal, Ander Ramos, Soumyadipta Acharya, Nitish V Thakor

**Affiliations:** 1 Department of Biomedical Engineering, The Johns Hopkins University, Baltimore, MD, USA; 2 Department of Biomedical Engineering, Fatronik Technological Foundation, Spain

## Abstract

**Background:**

It has been suggested that Brain-Computer Interfaces (BCI) may one day be suitable for controlling a neuroprosthesis. For closed-loop operation of BCI, a tactile feedback channel that is compatible with neuroprosthetic applications is desired. Operation of an EEG-based BCI using only *vibrotactile feedback*, a commonly used method to convey haptic senses of contact and pressure, is demonstrated with a high level of accuracy.

**Methods:**

A Mu-rhythm based BCI using a motor imagery paradigm was used to control the position of a virtual cursor. The cursor position was shown visually as well as transmitted haptically by modulating the intensity of a vibrotactile stimulus to the upper limb. A total of six subjects operated the BCI in a two-stage targeting task, receiving only vibrotactile biofeedback of performance. The location of the vibration was also systematically varied between the left and right arms to investigate location-dependent effects on performance.

**Results and Conclusion:**

Subjects are able to control the BCI using only vibrotactile feedback with an average accuracy of 56% and as high as 72%. These accuracies are significantly higher than the 15% predicted by random chance if the subject had no voluntary control of their Mu-rhythm. The results of this study demonstrate that vibrotactile feedback is an effective biofeedback modality to operate a BCI using motor imagery. In addition, the study shows that placement of the vibrotactile stimulation on the biceps ipsilateral or contralateral to the motor imagery introduces a significant bias in the BCI accuracy. This bias is consistent with a drop in performance generated by stimulation of the contralateral limb. Users demonstrated the capability to overcome this bias with training.

## Background

A Brain-Computer Interface (BCI) uses electrophysiological measures of brain activity to enable communication with external devices, such as computers and prostheses. Recent breakthroughs in the development of BCI have enabled practical applications that may help users with severe neuromuscular disabilities. By modulating changes in their electroencephalographic (EEG) activity, BCI users have demonstrated two-dimensional cursor control and the ability to type out messages on virtual keyboards [1-5].

A survey of individuals with upper-limb loss suggests that improving prosthetic control capabilities is a top priority in the community [6]. Most of these individuals are currently limited to cumbersome prostheses with myoelectric control or cable-operated systems and many in fact choose to avoid the hassle of a prosthesis [7,8]. It has been suggested that advances in BCI may eventually allow for control of neuroprostheses [9,10], with research groups already having demonstrated invasive cortical control of mechanical actuators in humans and nonhuman primates [11-13].

Of the numerous hardware and signal processing issues that must be resolved to make this goal a reality, one important factor which merits attention is the nature of the BCI biofeedback to the user. Conventional BCIs designed for the paralyzed have utilized a visual interface, such as a computer cursor or virtual keyboard, to close the control loop between the subject and the interface. While this modality is suitable for situations where the BCI user is interested in only the position and configuration of the controlled device, visual feedback is inadequate for grasping objects where haptic (relating to touch) senses such as grasping force are desired. To overcome this deficiency, a haptic information channel such as vibrotactile feedback can provide the user with the appropriate sensory information from a neuroprosthesis.

Vibrotactile feedback is a simple and compact mechanism commonly used in noninvasive haptic feedback systems because it is safe, straightforward to implement, and frees the user from having to maintain visual attention of the actuator [14]. Many vibrotactile feedback systems have been developed to convey information through a tactile interface when visual attention was deemed inefficient or unnecessary [15]. Prior prosthetics research also investigates how such feedback systems are used to convey the intensity of grasping force [16,17]. Since any advanced neuroprosthetic control will inevitably require communicating different haptic inputs to the user, the integration of haptic biofeedback to BCI applications deserves to be investigated.

This study uses a vibrotactile stimulus to provide one-dimensional feedback of a specific parameter, such as the output of a force sensor. The vibrotactile feedback is placed on the arm in order to mimic sensory stimulation provided on the residual limb of an amputee. Feedback at this location has been used in previous studies testing haptic feedback with upper-limb prostheses [18-20]. The BCI platform used to control this parameter is based on the modulation of Mu (8–12 Hz) rhythm activity via motor imagery tasks, which is a well-documented BCI control strategy [21-23]. Actual or imagined motor movements result in an event-related desynchronization (ERD) in spectral power at these frequencies over the sensorimotor cortex. Subjects can learn to modulate their Mu-band power to produce a 1-D control signal. The platform is designed to distinguish between three states: relaxation and two separable desynchronization patterns that are operant-conditioned from a starting baseline of right hand versus left hand motor imagery. This control paradigm can enable the *Open*, *Close*, and *Rest *commands needed to actuate an upper-limb prosthetic device in real time.

The goal of the study is to demonstrate that vibrotactile biofeedback is an effective method to enable closed-loop BCI control. This is a necessary step for the integration of a haptic information channel with a BCI-controlled prosthesis. Accuracy and latency statistics of BCI control using only vibrotactile biofeedback are presented to demonstrate the feasibility of the novel feedback approach. In addition, performance with vibrotactile feedback ipsilateral to hand motor imagery is compared to performance with feedback contralateral to hand motor imagery in order to determine whether the subjects' ability to modulate Mu rhythms is related to the location of the vibrotactile stimulus.

## Methods

### Experimental Setup

**Figure 1:**
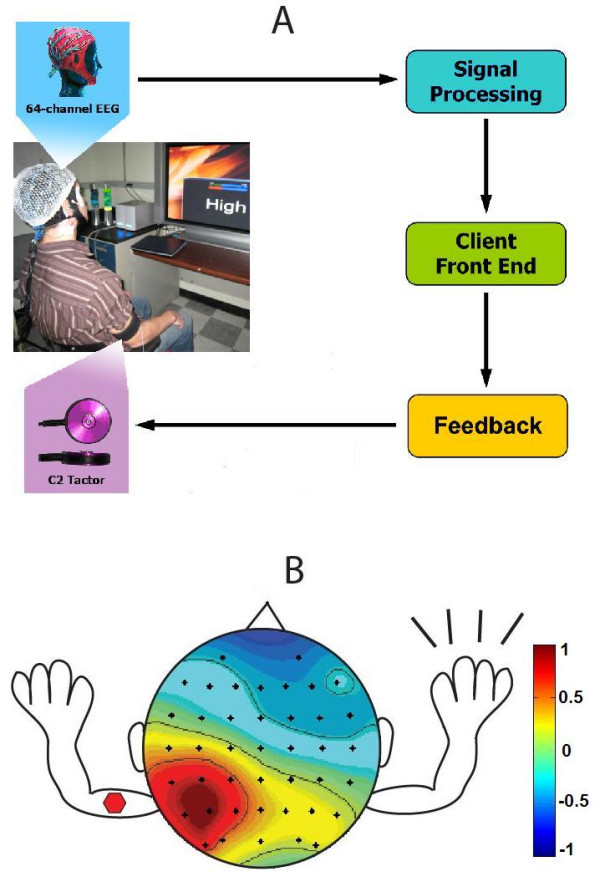
**Experimental Setup**. Experimental setup showing a closed-loop BCI system. A) 64-channel EEG data are acquired and used to control a BCI which returns state information to the user through vibrotactile feedback. B) Vibrotactile stimulation location is varied between limbs ipsilateral and contralateral to motor imagery (contralateral placement shown above). The scalp plot shows a representative independent component corresponding to right hand motor imagery.

Subjects used a three-state EEG-based BCI to control a parameter in one dimension (see Fig. 1a). Upon hearing an auditory cue of either *High *or *Low*, the subject would use the corresponding motor imagery task to move the parameter value to opposite levels. Two methods of feedback were supported for the BCI; 1) a visual interface that showed the parameter position on a horizontal bar on a monitor 3 ft from the subject and 2) a vibrotactile feedback system that conveyed the parameter state by modulating the pulse rate of a vibrating voice coil motor placed on the subject's arm. Subjects were trained with both the visual and vibrotactile interfaces simultaneously, and then moved to the vibrotactile interface only for data collection. The location of the vibration was also systematically varied between arms to investigate location-dependent effects on performance (see Fig. 1b).

**Figure 2:**
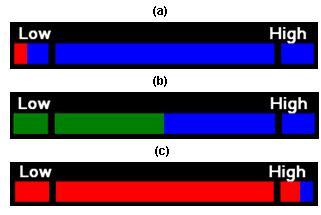
**Visual Interface**. Visual interface displaying horizontal bar that is proportional to level of vibrotactile feedback. A) shows bar when *Low *cue is reached (Level 1) successfully, B) shows bar at beginning of each trial (Level 4), and C) shows bar when *High *cue is reached (Level 7).

For *High *cues, the subject used right hand motor imagery to increase the frequency of vibration to the maximum level (Level 7), whereas for *Low *cues the subject used left hand motor imagery to decrease the frequency of vibration to the minimum level (Level 1). Each trial began at a mid-level vibration (Level 4) that did not correspond to either *Low *or *High*, and the subject failed the task if they remained in this region (Level 2–6). The visual interface shown in Fig. 2 mirrored the vibrotactile stimulus, incrementing or decrementing in 7 discrete levels, and with *Low *and *High *target endpoints at the extreme left and right respectively.

A total of six healthy male adults (aged 21–25) participated in the study. Subjects A, B, D and F had no previous BCI training, while Subjects C and E had 25 and 12 hours of previous BCI training respectively. Informed consent was obtained from all subjects, and all data were collected under certification from the Johns Hopkins University Institutional Review Board.

### EEG Data Acquisition

EEG was acquired using a Neuroscan SynAmps^2 ^64-channel amplifier from Compumedics (El Paso, TX). A QuickCap 64-channel EEG cap (modified 10–20 system) from Neuroscan was used for data acquisition; referenced between Cz and CPz, and grounded anteriorly to Fz.

The SynAmps^2 ^amplifier and signal processing modules were connected through client-server architecture, with the amplifier acting as the server and the signal processing module running on a stand-alone client PC. Data were sampled at 250 Hz and transmitted over a TCP/IP protocol to the client PC for storage and real-time signal processing using a custom BCI platform.

### Mu-Band Extraction with Hierarchical Classifiers

The control signal output by the BCI was based on extracting peak Mu-band power, which is well known to be modulated by motor imagery [21-23]. In general, the EEG activity for right hand and left hand motor imagery were focused at electrodes C3 and C4, respectively, which overlay the M1 hand area [24]. A large Laplacian spatial filter was applied by re-referencing each electrode to the mean of its next-nearest neighboring electrodes [25].

The spatially filtered EEG activity from each electrode was modeled as an autoregressive (AR) process over a sliding temporal window of duration *T*_*W *_s shifting every *T*_*S *_s,

y[n]=∑k=0Kaky[n−k]+ε[n]
 MathType@MTEF@5@5@+=feaafiart1ev1aaatCvAUfKttLearuWrP9MDH5MBPbIqV92AaeXatLxBI9gBaebbnrfifHhDYfgasaacH8akY=wiFfYdH8Gipec8Eeeu0xXdbba9frFj0=OqFfea0dXdd9vqai=hGuQ8kuc9pgc9s8qqaq=dirpe0xb9q8qiLsFr0=vr0=vr0dc8meaabaqaciaacaGaaeqabaqabeGadaaakeaacqWG5bqEcqGGBbWwcqWGUbGBcqGGDbqxcqGH9aqpdaaeWbqaaiabdggaHnaaBaaaleaacqWGRbWAaeqaaOGaemyEaKNaei4waSLaemOBa4MaeyOeI0Iaem4AaSMaeiyxa0faleaacqWGRbWAcqGH9aqpcqaIWaamaeaacqWGlbWsa0GaeyyeIuoakiabgUcaRGGaciab=v7aLjabcUfaBjabd6gaUjabc2faDbaa@4AD1@

where *a*_*k *_were the autoregressive coefficients, *K *was the model order, and *ε[n] *was an independent identically distributed stochastic sequence with zero mean and variance *σ*^2 ^[26]. *T*_*W *_and *T*_*S *_were typically chosen to be 2 s and 250 ms, respectively, with a model order *K *of 12–15. Model orders above this range have been shown to yield minimal improvements in regression accuracy of the sensorimotor rhythm [27]. Burg's method [28] was used to estimate the time-varying AR coefficients.

The power spectral density (in dB) of the AR process for each electrode was then computed as,

*P*(*ω*) = 10 log(*h*(*ω*))

where h(ω)=σ21+|a1e−iω+...+aKe−iKω|2
 MathType@MTEF@5@5@+=feaafiart1ev1aaatCvAUfKttLearuWrP9MDH5MBPbIqV92AaeXatLxBI9gBaebbnrfifHhDYfgasaacH8akY=wiFfYdH8Gipec8Eeeu0xXdbba9frFj0=OqFfea0dXdd9vqai=hGuQ8kuc9pgc9s8qqaq=dirpe0xb9q8qiLsFr0=vr0=vr0dc8meaabaqaciaacaGaaeqabaqabeGadaaakeaacqWG3bWDcqWGObaAcqWGLbqzcqWGYbGCcqWGLbqzcqqGGaaicqWGObaAcqGGOaakiiGacqWFjpWDcqGGPaqkcqGH9aqpdaWcaaqaaiab=n8aZnaaCaaaleqabaGaeGOmaidaaaGcbaGaeGymaeJaey4kaSYaaqWaaeaacqWGHbqydaWgaaWcbaGaeGymaedabeaakiabdwgaLnaaCaaaleqabaGaeyOeI0IaemyAaKMae8xYdChaaOGaey4kaSIaeiOla4IaeiOla4IaeiOla4Iaey4kaSIaemyyae2aaSbaaSqaaiabdUealbqabaGccqWGLbqzdaahaaWcbeqaaiabgkHiTiabdMgaPjabdUealjab=L8a3baaaOGaay5bSlaawIa7amaaCaaaleqabaGaeGOmaidaaaaaaaa@5919@

and the peak mu-band power was extracted at discrete times *t*_*k*_,

*P*_*C*3_(*t*_*k*_) = max(*P*_*C*3_(*ω*_*μ*_))

*P*_*C*4_(*t*_*k*_) = max(*P*_*C*4_(*ω*_*μ*_))

where *ω*_*μ *_is the frequency range of the mu-band (8–12 Hz).

A novel two-stage hierarchical linear classification scheme was used to generate the final output control signal. A gating classifier *G *was designed to distinguish between motor imagery ERD and relaxation,

G(tk)={1if w1GPC3(tk)+w2GPC4(tk)+BG<TG0else
 MathType@MTEF@5@5@+=feaafiart1ev1aaatCvAUfKttLearuWrP9MDH5MBPbIqV92AaeXatLxBI9gBaebbnrfifHhDYfgasaacH8akY=wiFfYdH8Gipec8Eeeu0xXdbba9frFj0=OqFfea0dXdd9vqai=hGuQ8kuc9pgc9s8qqaq=dirpe0xb9q8qiLsFr0=vr0=vr0dc8meaabaqaciaacaGaaeqabaqabeGadaaakeaacqWGhbWrcqGGOaakcqWG0baDdaWgaaWcbaGaem4AaSgabeaakiabcMcaPiabg2da9maaceaabaqbaeaabiGaaaqaaiabigdaXaqaaiabdMgaPjabdAgaMjabbccaGiabdEha3jabigdaXmaaBaaaleaacqWGhbWraeqaaOGaemiuaa1aaSbaaSqaaiabdoeadjabiodaZaqabaGccqGGOaakcqWG0baDdaWgaaWcbaGaem4AaSgabeaakiabcMcaPiabgUcaRiabdEha3jabikdaYmaaBaaaleaacqWGhbWraeqaaOGaemiuaa1aaSbaaSqaaiabdoeadjabisda0aqabaGccqGGOaakcqWG0baDdaWgaaWcbaGaem4AaSgabeaakiabcMcaPiabgUcaRiabdkeacnaaBaaaleaacqWGhbWraeqaaOGaeyipaWJaemivaq1aaSbaaSqaaiabdEeahbqabaaakeaacqaIWaamaeaacqWGLbqzcqWGSbaBcqWGZbWCcqWGLbqzaaaacaGL7baaaaa@5EB5@

where *w1*_*G*_, *w2*_*G*_, *B*_*G*_, and *T*_*G *_were the weights, bias, and threshold, respectively, determined online for each subject. A second movement classifier *M *was designed to distinguish between right hand and left hand motor imagery tasks,

M(tk)={+1if w1MPC3(tk)+w2MPC4(tk)+BM<TM−1else
 MathType@MTEF@5@5@+=feaafiart1ev1aaatCvAUfKttLearuWrP9MDH5MBPbIqV92AaeXatLxBI9gBaebbnrfifHhDYfgasaacH8akY=wiFfYdH8Gipec8Eeeu0xXdbba9frFj0=OqFfea0dXdd9vqai=hGuQ8kuc9pgc9s8qqaq=dirpe0xb9q8qiLsFr0=vr0=vr0dc8meaabaqaciaacaGaaeqabaqabeGadaaakeaacqWGnbqtcqGGOaakcqWG0baDdaWgaaWcbaGaem4AaSgabeaakiabcMcaPiabg2da9maaceaabaqbaeaabiGaaaqaaiabgUcaRiabigdaXaqaaiabdMgaPjabdAgaMjabbccaGiabdEha3jabigdaXmaaBaaaleaacqWGnbqtaeqaaOGaemiuaa1aaSbaaSqaaiabdoeadjabiodaZaqabaGccqGGOaakcqWG0baDdaWgaaWcbaGaem4AaSgabeaakiabcMcaPiabgUcaRiabdEha3jabikdaYmaaBaaaleaacqWGnbqtaeqaaOGaemiuaa1aaSbaaSqaaiabdoeadjabisda0aqabaGccqGGOaakcqWG0baDdaWgaaWcbaGaem4AaSgabeaakiabcMcaPiabgUcaRiabdkeacnaaBaaaleaacqWGnbqtaeqaaOGaeyipaWJaemivaq1aaSbaaSqaaiabd2eanbqabaaakeaacqGHsislcqaIXaqmaeaacqWGLbqzcqWGSbaBcqWGZbWCcqWGLbqzaaaacaGL7baaaaa@60C2@

where *w1*_*M*_, *w2*_*M*_, *B*_*M*_, and *T*_*M *_are the weights, bias, and threshold, respectively, determined online for each subject. The final output *F(t_k_) *was the product of the two classifiers,

*F*(*t*_*k*_) = *G*(*t*_*k*_) × *M*(*t*_*k*_)

where +1 corresponds to right-hand movement, -1 to left-hand movement, and 0 to relaxation. A classifier decision was made every 250 ms.

This 3-type classification is highly appropriate for prosthetic applications, where a user controlling a prosthetic device will require an easily-maintained "rest" state. This is achieved with a gating classifier. Only when the subject is actively trying to produce a movement (e.g. open or close a prosthetic hand) does the movement classifier distinguish the movement type.

### Vibrotactile Feedback System

Vibratory feedback was provided by a C2 voice coil tactor from Engineering Acoustics, Inc. (Winter Park, FL), which was placed on the biceps with an elastic cuff. Feedback at this location has been used in previous studies testing haptic feedback with prosthetic technology [18-20]. Furthermore, psychophysical responses to stimulation in this location have been well-characterized [29].

The vibratory stimulus waveform was a series of discrete pulses with a fixed duty cycle of 50%. The waveform was modulated by varying the width of the pulses to change the pulse rate. Shorter, more rapid pulses were perceived as an increase in stimulus intensity, and longer, less rapid pulses were perceived as a decrease in stimulus intensity. The vibration carrier frequency for each pulse was 200 Hz in order to maximally stimulate high-frequency Pacinian mechanoreceptors [30].

The range of vibration waveforms comprised of 7 discrete pulse rates. A BCI classifier output of +1 generated by right hand motor imagery increased the pulse rate, while a classifier output of -1 generated by left hand motor imagery decreased the pulse rate. A classifier output of 0, implying relaxation, kept the pulse rate constant. All cues and success/failure indicators were presented to the subject audibly through headphones. In addition, to ensure that the subject was responding to purely the tactile sensation, the headphones played white noise throughout the trial that masked any audible vibrations from the tactor.

### Subject Training

Each subject underwent a training period at the beginning of the study in order to determine the thresholds for the gating classifier and movement classifier. During this time the subject practiced right and left hand motor imagery tasks to modulate his Mu rhythm while the classifier parameters were optimized. For each classifier, the thresholds were set halfway between the average mu rhythm powers for the two separable states. These values were set manually for each subject using a utility that allowed the operator to visualize and adjust the parameters online. Once the optimal weights and biases were selected during this training period, they remained constant for the duration of the study for that subject. Total training time varied due to subject to subject learning variations (ranging from 10 min. for experienced Subject D to 30 min. for novice Subject A). After the final optimization, the subject was allowed to rest for 5 min prior to the start of the study.

### Study Design: BCI Control of Vibrotactile Stimulus

**Figure 3:**
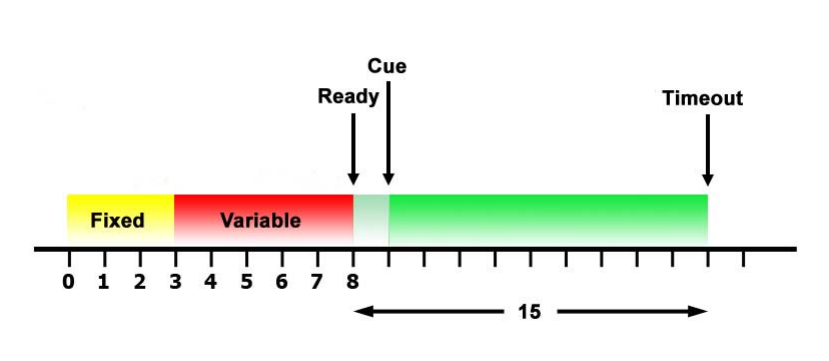
**Trial Timing Diagram**. Timing diagram for each trial. Each trial starts with a variable 3–8 s rest period, followed by an auditory *Ready *cue. After 1 s, an auditory *High *or *Low *cue is given. The maximum length of each trial is 15 s.

The task was designed to test the subject's ability to operate a BCI to control the strength of a vibrotactile stimulus. As shown in the timing diagram in Fig. 3, each experimental trial began with a variable 3–8 s rest period, at the end of which the subject was presented with an auditory *Ready *cue. Following the *Ready *period of 1 s, either a *Low *or *High *cue was given to the subject audibly. The cues were provided through the headphones and overlaid the white noise. The trial ended successfully if the subject reached the intended vibration level and maintained this position for 1 s. The trial ended with failure in two ways: 1) failure at timeout if the subject could not complete the task in 15 s and 2) immediate failure if the subject reached and maintained the incorrect vibration level for 1 s.

A single recording session consisted of a training period and a testing period. During the training period, the subject performed a variable number of training sets; each consisting of 10 trials with five *High *and five *Low *cues presented in a pseudorandom order. These training sets were performed with both visual feedback and a *constant *level of vibrotactile stimulation on the right biceps. In this phase of the experiment, the vibrotactile stimulation did not convey any information, but was present to acclimatize the subject to the conditions of the testing period. Subjects completed multiple training sets until they achieved a success rate of 60% – at which point they moved on to the testing period.

During the testing period, the subject completed six trial sets; each consisting of 20 trials with 10 *High *and 10 *Low *cues presented in a pseudorandom order. The first two testing sets were performed with both visual feedback and vibrotactile feedback so the subject could map changes in the vibrotactile stimulation to the visual display. The position of the tactor was varied between trial sets so that the feedback alternated between left and right arm. The remaining four testing sets were performed with only vibrotactile feedback (and alternating tactor placement). The entire recording session ran for approximately 2 hours, including 2 minute breaks between trial sets and additional break time as needed.

## Results

### Performance Measures for BCI

Accuracy was defined as the percentage of trials where the subject completed the BCI control task successfully. Latency was defined as the time required to complete the task successfully. Accuracy and latency results for vibrotactile feedback trials are reported in Table 1 for each subject, separated by trials where the tactor was placed ipsilateral or contralateral to the motor imagery.

**Table 1:** BCI Performance Results. Accuracy and latency results are reported for each subject, separated by trials where tactor was placed ipsilateral or contralateral to the motor imagery. Accuracies for trials with ipsilateral placement are generally higher than accuracies for trials with contralateral placement.

	ACCURACIES		LATENCIES	
SUBJECT ID	Ipsilateral	Contralateral	Ipsilateral (s)	Contralateral (s)

A	65%	70%	8.58	7.62
B	48%	30%	8.93	9.86
C	53%	27%	8.15	8.14
D	64%	57%	7.68	7.40
E	86%	58%	8.46	7.57
F	70%	50%	6.80	8.89

**Figure 4:**
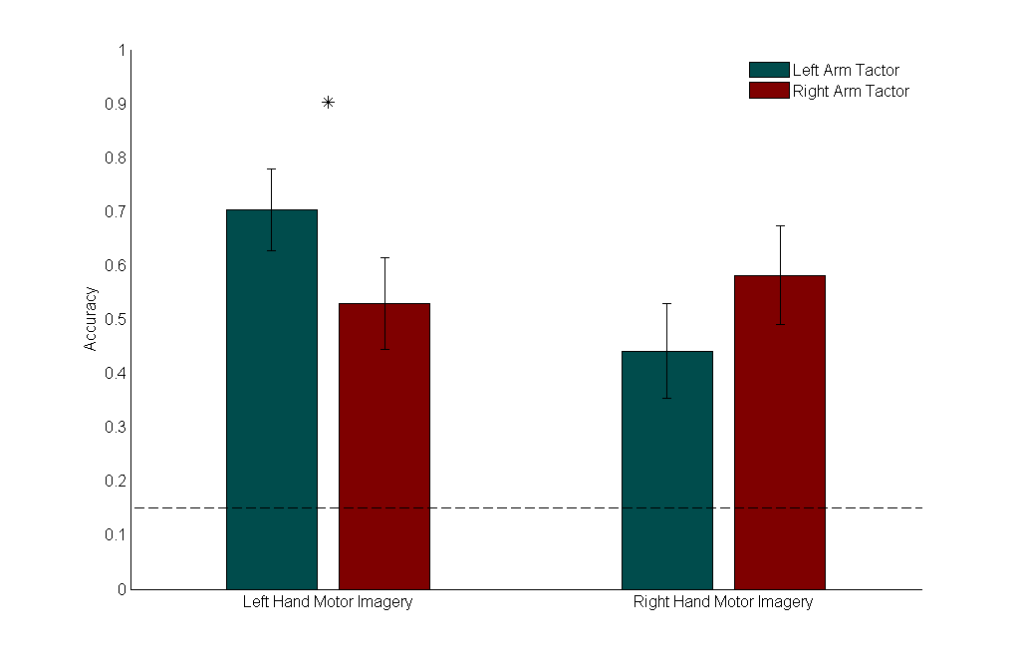
**Accuracy Comparison**. Means and standard errors of accuracies across all subjects, separated by motor imagery and tactor location. The dotted line indicates the success rate expected through random chance (15%). For *Low *cues (which required left hand motor imagery), mean accuracy was statistically significantly higher with vibratory stimulus on the left arm (*p *= 0.031). For *High *cues (which required right hand motor imagery), mean accuracy was higher with the stimulus on the right arm.

Accuracy statistics were calculated for trials where the subject received only vibrotactile feedback. The average accuracy results across all subjects, separated by both motor imagery and tactor placement, are presented in Fig. 4. The data show that on average, subject accuracy was 56%, which was significantly larger than the probability of randomly achieving success, as outlined below.

Due to the use of a three-state classifier, and the fact that the subject must maintain the *Low *or *High *vibration level for 1 s, the probability of randomly succeeding was 15%. Since a classifier decision was made every 250 ms and the timeout period for each trial was 15 s, there was a maximum of 60 classification outputs per trial. The user began each trial from a mid-vibration level, and seven consecutive outputs of +1(-1) were needed to reach the maximum(minimum) vibration level. To successfully complete the trial, the user then had to maintain the correct vibration level for an additional 1 s, or four classification outputs. Therefore, the fastest a user could complete a trial was 2.75 s. Assuming a 1:1 classification distribution between 0/+1 for the gating classifier, and a 1:1 classification distribution between +1/-1 for the movement classifier, a random walk over 10,000 simulated trials yielded an average success rate of 15%.

Fig. 4 also suggests that the accuracy for particular cues varied with tactor placement. Tests for significant difference in medians between left arm and right arm accuracies were performed using the Wilcoxon Sign Rank test. During trials with a *Low *cue (which required left hand motor imagery), average performance was significantly better with the tactor on the left biceps (*p *= 0.031). During trials with a *High *cue (which required right hand motor imagery), the average performance was better with the tactor on the right biceps, although the increase was not statistically significant (*p *= 0.150). The general trend appears to be that the vibrotactile stimulus biases results in favor of the outcome requiring motor imagery of the hand ipsilateral to the tactor location.

**Figure 5:**
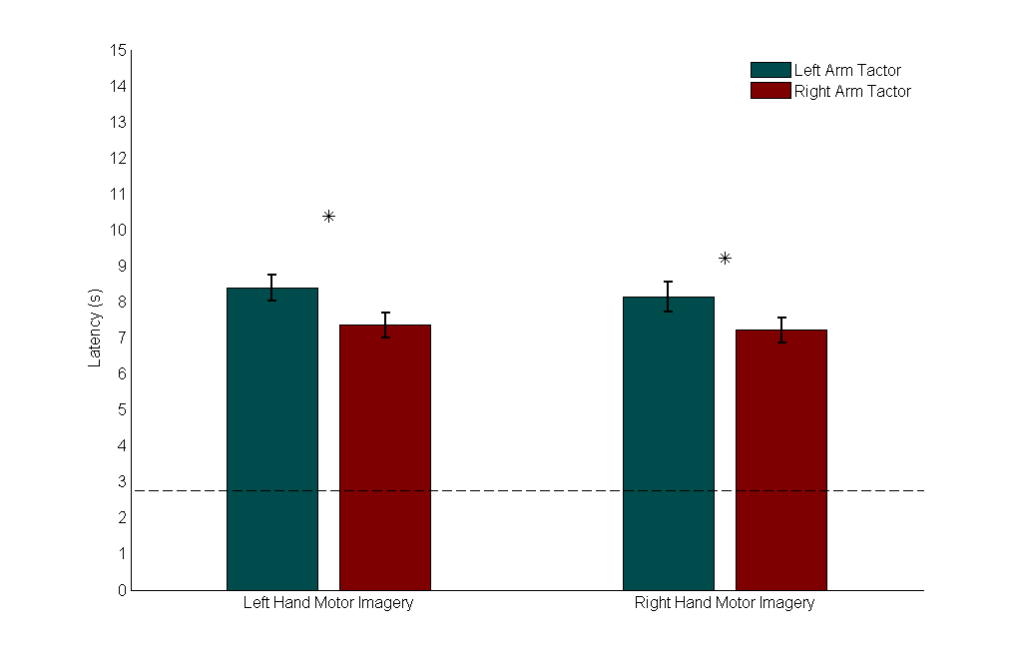
**Latency Comparison**. Means and standard errors for average latencies across all subjects, separated by motor imagery and tactor location. The lower dotted line indicates the fastest possible trial time (2.75 s) while the upper dotted line indicates the trial timeout value (15 s). For *Low *cues (which required left hand motor imagery), mean latency was statistically significantly longer by 1.04 s with vibratory stimulus on the left arm (*p *= 0.046). For *High *cues (which required right hand motor imagery), mean latency was again statistically significantly longer by 0.92 s with vibratory stimulus on the left arm (*p *= 0.033).

Latency statistics were also computed for the trials where the subject received only vibrotactile feedback. The average latency results across all subjects, separated by both motor imagery and tactor placement, are presented in Fig. 5. A comparison of medians using the Mann-Whitney U test shows that during trials with a *Low *cue (which required left hand motor imagery), average latencies were significantly longer by 1.04 s with the tactor on the left biceps (*p *= 0.046). Similarly, during trials with a *High *cue (which required right hand motor imagery), average latencies were significantly longer by 0.92 s with again the tactor on the left biceps (*p *= 0.033).

**Figure 6:**
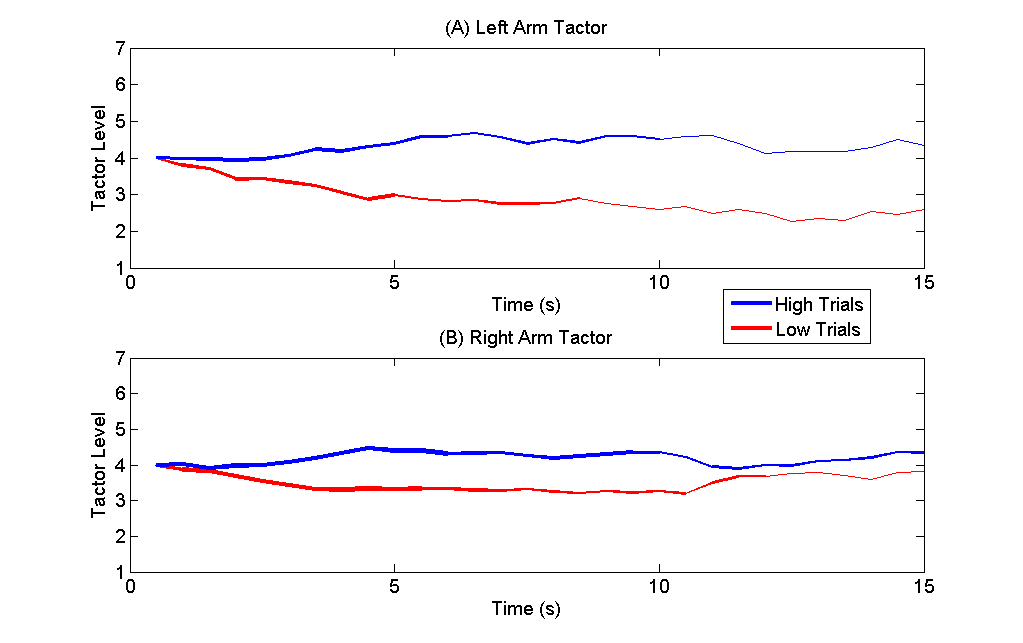
**Trajectory Comparison**. Mean trajectory plot for all subjects with A) tactor placed on left arm, and B) tactor placed on right arm. The mean trajectory of *High *trials (which required right hand motor imagery) is shown in blue while the mean trajectory of *Low *trials (which required left hand motor imagery) is shown in red. The thickness of the line is proportional to the number of trials. Faster divergence and clearer separation is evident between *Low *and *High *trajectories when tactor is on the left arm.

Trajectory plots were generated to visualize the subjects' control throughout the duration of the trial. A mean trajectory plot for all trials with the tactor placed on the left arm is shown in Fig. 6a, and with the tactor placed on the right arm in Fig. 6b. Since the trajectory duration for each trial varied with subject performance, the thickness of the mean trajectory is drawn proportional to the number of trials that reached that length of time (this value drops with time due to early successes and failures). The mean trajectory is shown in blue for trials with a *High *cue (which required right hand motor imagery) and in red for trials with a *Low *cue (which required left hand motor imagery). Trials with the tactor on the left arm (Fig. 6a) showed faster divergence and a clearer separation between *Low *and *High *mean trajectories.

### EEG Data Analysis

**Figure 7:**
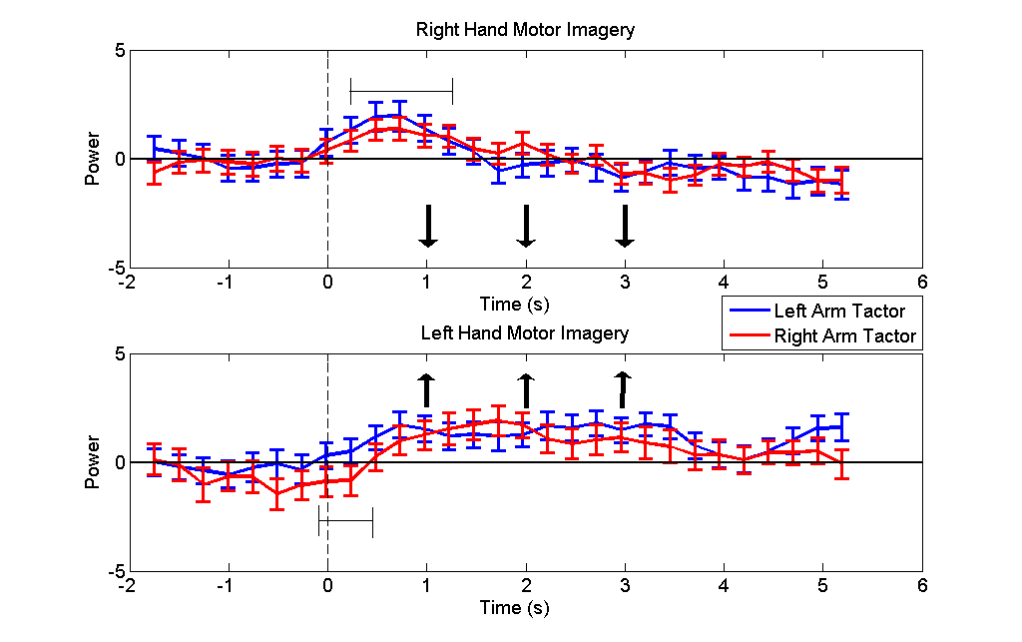
**Mu Band Power**. Plot of the difference in peak Mu-band power between electrodes C3 and C4, averaged across all subjects and trials and separated by tactor location. A) shows data from right hand motor imagery trials (*High *cues), and B) shows data from left hand motor imagery trials (*Low *cues). The direction for the desired motor imagery task is indicated with arrows. Horizontal bars show where tactor placement produced noticeable deviations in the control signal early on.

In addition to performance statistics, the peak Mu-band powers from electrodes C3 (*P*_*C*3_) and C4 (*P*_*C*4_) were recorded for all subjects and analyzed using EEGLAB v. 5.02 (Schwartz Center for Comp. Neurosci., UCSD, CA) [31]. Since the movement classifier accepts the weighted difference of these values (see Eq. 6), a plot of (*P*_*C*3 _-*P*_*C*4_) characterizes the subjects' Mu-band activity and allows for the separation of left and right hand motor imagery patterns. These plots were averaged across all trials and subjects. The cumulative plot with standard error bars is shown in Fig. 7. The results for right hand motor imagery trials (*High *cues) are shown in Fig. 7a and the results for left hand motor imagery trials (*Low *cues) are shown in Fig. 7b.

Fig. 7 shows that tactor placement tended to disturb the control signal early on in the trial, but that this influence was reduced as the trial progressed. Contralateral placement showed greater deviation from ipsilateral placement in left arm tactor trials, indicating a greater separation in performance in left arm trials, which is consistent with the trajectory analysis. In general, the contralateral and ipsilateral traces merged as the trial progressed, indicating that the tactor bias effects weakened as the trial progressed and the user compensated for the vibrotactile stimulation.

## Discussion

### BCI Feedback Represents Haptic Information

To successfully complete our task, the subject was required to drive a parameter from an initial medium state to either a low or a high state and maintain it for 1 s. The low and high states represented discrete regions of a 2-D space with a third neutral state between them. The rationale for selecting this type of task is based on the application of a BCI to the context of upper-limb prosthetics. The primary motivation for pursuing vibrotactile biofeedback is to develop a method whereby haptic information can be provided to the user in an appropriate manner. One can imagine a BCI user controlling an advanced neuroprosthesis to grasp an object. Just as robotic mechanisms in teleoperation systems transmit forces from the end-effector to the operator, this advanced prosthesis is instrumented with force sensors so that force information can be transmitted to the user. A compact and safe vibrotactile feedback system is used to convey this force information and as a result, the BCI operator's ability to interpret and modulate his grasping force is improved.

With this application in mind, the appropriate BCI task is not the selection of a particular state as in a hierarchical selection tree, but rather the direct control of a certain parameter whose state is conveyed through biofeedback. If the vibrotactile intensity is thought to represent grip force strength, then the task of driving the intensity high (through right hand motor imagery) may be thought of as squeezing a grasped object while driving the intensity low (through left hand motor imagery) would represent releasing the object. Furthermore, maintaining a constant intensity level (through relaxation) would be equivalent to maintaining a steady hold on the object. The development of a three-state, self-paced BCI based on simple motor movement was motivated by this intended neuroprosthesis control paradigm and proved sufficient to test the efficacy of a vibrotactile feedback system. It should be noted that more complex BCIs that operate using different control paradigms may interact with haptic stimuli differently.

### Establishing BCI Performance Capability

Accuracy and latency statistics are the preferred methods in literature for quantifying the performance of a BCI [32-34]. However, due to the nature of our defined task, performance figures from this study should not be compared to results from BCIs designed for different purposes. Unlike a typical two-state selection paradigm, the random chance of success is not 50%, but actually much lower due to the difficulty of the task as described in the previous section. The effectiveness of this control scheme is established by demonstrating that accuracies across all cues and tactor locations were significantly higher than the 15% random chance of success.

The accuracies from the vibrotactile feedback trials demonstrate that vibrotactile stimulation is an effective means to provide feedback information in 1-D. Considering the fact that four of the six subjects had no prior BCI experience, additional training sessions would likely improve performance further, as expected with any BCI paradigm. The learning process for this feedback modality was facilitated by the study protocol, which was designed to introduce the vibrotactile biofeedback by associating it with a commonly used visual feedback system. The sequential process of training the subject with visual feedback, mapping the visual feedback to the vibrotactile feedback, and then finally testing with vibrotactile feedback, allowed the subject to mentally associate the different stimulus modalities. This type of paired stimulus presentation has been used successfully in prior studies of haptic feedback training [35,36] and studies of associative learning [37].

### Tactor Placement Bias

**Figure 8:**
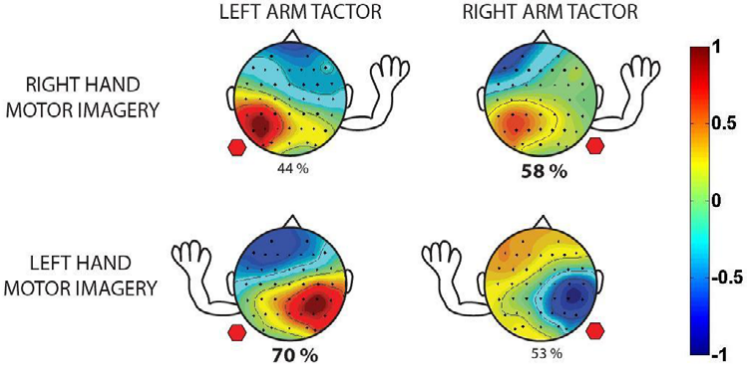
**Summary Accuracy Comparison**. This representative diagram shows summary accuracy values, separated by motor imagery type and tactor location. The location of the arm shows the motor imagery type (either right hand or left hand) and the location of the hexagon indicates the location of the tactor (right arm or left arm). Success at a motor imagery task was higher when the tactor was placed on the ipsilateral arm. Scalp plots show representative independent components corresponding to the respective motor imagery task.

The accuracy data also indicated that a significant bias was introduced with regards to the tactor placement location. Left arm tactor placement led to better performance for *Low *cues and right arm tactor placement led to better performance for *High *cues. Since *Low *and *High *were mapped to left and right hand imagery respectively, it appears that the tactor bias is consistent with either an enhancement of Mu rhythm desynchronization from ipsilateral hand imagery or an inhibition of Mu rhythm desynchronization from contralateral hand imagery. These results are summarized in Fig. 8.

The offline analysis of EEG data suggests that the latter case is true. The plots of the difference in peak Mu-band power from between C3 and C4 show that, on average, contralateral vibrotactile stimulation produces deviations in the signal in the first second of the trial. The contralateral and ipsilateral average traces eventually converge, indicating that subjects were able to overcome the vibratory influence to an extent. If so, the tactor bias is under some level of voluntary control and may be mitigated with greater concentration and training time. This hypothesis is supported by impressions from subjects who noted that vibrotactile feedback tended to draw attention to the stimulated hand. Although this inadvertent attention might lead to changes similar to those associated with motor imagery, most subjects reported that they were able to consciously re-focus on the required motor imagery task while maintaining their awareness of the information from the vibrotactile feedback.

The mean trajectory plots suggest that average subject performance is different for tactor placement on the left arm versus the right arm, as evidenced by a higher rate of divergence and earlier point of separation between *Low *and *High *trials for mean left arm trajectories. This could be due to a combination of a) faster successes during *Low *trials leading to the early divergence, and b) faster failures during *High *trials which keep the average trajectories separate at later stages of the trial. These results are supported by the accuracy data, which show that on average, ipsilateral left arm *Low *trials were the most accurate (70%) while contralateral left arm *High *trials were the least accurate (44%).

While a significant disparity exists between ipsilateral and contralateral motor imagery accuracies with the tactor on the left arm, the disparity is muted with the tactor on the right arm (58% for ipsilateral vs. 53% for contralateral). Furthermore, the average latency of trials with the tactor on the left arm was 0.98 s longer than trials with the tactor on the right arm with a high statistical significance. It is possible that the training protocol of acclimating our subjects with right arm tactor stimulation may have led them to better adapt to motor imagery tasks with the feedback at this location. It should also be noted that the tactor bias results are averaged from only two experienced subjects and four novice subjects. It remains to be seen whether sufficient training with the vibrotactile stimulus at alternate locations can reduce the difference in performance between ipsilateral and contralateral motor imagery tasks.

The bias effect may be mitigated through training as well as modifications to the BCI signal processing. Adjusting the thresholds and weights for the linear classifier appropriately, possibly by introducing an adaptive algorithm, could compensate for the stimulation and reduce this bias. Adaptive algorithms have been utilized in some of the latest BCIs to improve robustness against changes in brain dynamics brought about by fatigue and other factors [3,38]. These methods adjust the weights and biases of the classifiers on a trial-by-trial basis by using optimization algorithms such as Least-Mean-Squares method [23]. Further work will be needed to determine if similar methods can adapt to the vibrotactile stimulation during real-time BCI classification.

## Conclusion

A tactile information channel will be a critical component of any BCI designed to control an advanced neuroprosthetic device. To test the efficacy of this approach, a motor imagery BCI was enhanced with a vibrotactile feedback channel to convey 1-D information. A hierarchical classification scheme was used to generate output appropriate for prosthesis grasping tasks. Subjects were initially trained to perform BCI control tasks with a visual feedback system and were then migrated to the vibrotactile feedback system. The performance results show that all subjects were able to operate a three-state motor imagery BCI using only vibrotactile biofeedback, but that variations in tactor placement led to a notable bias in accuracy. The EEG data indicate that the choice of vibrotactile stimulus location biased the user's modulation of Mu-rhythm activity towards desynchronization generated by imagery of the ipsilateral hand. Analysis of latency and trajectory data indicate that preferences for stimulation location may be affected by the training protocol. Further work will be needed to determine exactly how the neural correlates of vibrotactile feedback affect the modulation of Mu-rhythm activity and to determine optimal signal processing techniques to account for the feedback. In conclusion, the successful incorporation of training procedures and mechanisms to compensate for vibrotactile feedback bias in BCI platforms will aid the development of haptic biofeedback systems for neuroprosthetic and other applications.

## Authors' contributions

AC participated in study design, running the experiments, data analysis, and drafted the manuscript. VA participated in running the experiments, data analysis, and drafted the manuscript. AR participated in running the experiments and data analysis. SA participated in study design and running the experiments. NVT participated in study design and supervised the research. All authors read and approved of the final manuscript.
